# Comparison of myofibrillar protein degradation, antioxidant profile, fatty acids, metmyoglobin reducing activity, physicochemical properties and sensory attributes of *gluteus medius* and *infraspinatus* muscles in goats

**DOI:** 10.1186/s40781-016-0105-5

**Published:** 2016-06-15

**Authors:** Kazeem D. Adeyemi, Rafiat M. Shittu, Azad B. Sabow, Ahmed A. Abubakar, Roselina Karim, Saiful A. Karsani, Awis Q. Sazili

**Affiliations:** Department of Animal Science, Faculty of Agriculture, Universiti Putra Malaysia, 43400 UPM Serdang, Selangor Malaysia; Department of Food Technology, Faculty of Food Science and Technology, Universiti Putra Malaysia, 43400 UPM Serdang, Selangor Malaysia; Halal Products Research Institute, Universiti Putra Malaysia, 43400 UPM Serdang, Selangor Malaysia; Laboratory of Animal Production, Institute of Tropical Agriculture, Universiti Putra Malaysia, 43400 UPM Serdang, Selangor Malaysia; Department of Animal Production, University of Ilorin, PMB 1515 Ilorin, Nigeria; Department of Animal Resource, University of Salahaddin, Erbil, Kurdistan Region Iraq; Institute of Biological Sciences, Faculty of Science, University of Malaya, Kuala Lumpur, Malaysia

**Keywords:** Actin, Antioxidants, Carbonyl, Myosin, Oxidation, Sensory

## Abstract

**Background:**

The functionality of myofibrillar proteins is a major factor influencing the quality attributes of muscle foods. Nonetheless, the relationships between muscle type and oxidative changes in chevon during ageing are meagrely elucidated. *Postmortem* changes in antioxidant status and physicochemical properties of glycolytic *gluteus medius* (GM) and oxidative *infraspinatus* (IS) muscles in goats were compared.

**Methods:**

Twenty Boer bucks (9–10 months old, body weight of 36.9 ± 0.725 kg) were slaughtered and the carcasses were subjected to chill storage (4 ± 0.5 °C). Analyses were conducted on GM and IS muscles sampled on 0, 1, 4 and 7 d *postmortem*.

**Results:**

Chill storage did not affect the antioxidant enzyme activities in both muscles. The IS had greater (*P* < 0.05) superoxide dismutase and catalase activities than GM. Carotenoid and tocopherol contents did not differ between muscles but decreased (*P* < 0.05) over storage. The IS had higher (*P* < 0.05) glycogen and ultimate pH and lower (*P* < 0.05) shear force and cooking loss than GM. The carbonyl content, % metmyoglobin, drip loss and TBARS increased (*P* < 0.05) while free thiol, metmyoglobin reducing activity (MRA), shear force and myoglobin decreased (*P* < 0.05) over storage. Muscle type had no effect (*P* > 0.05) on free thiol, MRA and TBARS. The GM had lower (*P* < 0.05) redness on d 0 and 1 than IS while the IS had greater carbonyl, % metmyoglobin and drip loss than GM on d 7. The reflective density of slow myosin heavy chain (MHC) was higher (*P* < 0.05) while the density of fast MHC and actin was lower (*P* < 0.05) in IS than GM. Regardless of muscle type, the density of MHC decreased (*P* < 0.05) while that of actin was stable over storage. Nonetheless, the degradation of fast and slow MHC was greater (*P* < 0.05) in IS than GM. Muscle type had no effect (*P* > 0.05) on consumer preference for flavour, juiciness and overall acceptability. However, IS had higher (*P* < 0.05) tenderness score than GM on d 1 and 4 *postmortem*. Intramuscular fat was higher (*P* < 0.05) in IS compared with GM. Fatty acid composition did not differ between the muscles. However, GM had lower (*P* < 0.05) n-6/n-3 ratio than IS. The n-3 and n-6 PUFA declined (*P* < 0.05) while the SFA increased (*P* < 0.05) over storage.

**Conclusion:**

The changes in myofibrillar proteins and physicochemical properties of goat meat during *postmortem* chill storage are muscle-dependent.

## Background

*Postmortem* changes in muscle and its conversion to meat play an important role in meat quality [1]. *Postmortem* ageing is a common practice in the red meat industry. Ageing as used in this context, refers to the act of holding meat post-rigor at refrigeration temperatures for a given duration in order to improve meat quality traits, especially tenderness, and to supply chilled meat to distance markets [2]. Although the efficacy of *postmortem* ageing in achieving the aforementioned aims has been underscored [1, 2], *postmortem* ageing could have negative impact on the oxidative stability of lipids, myoglobin and myofibrillar proteins in meat [3–5]. Oxidative deterioration of lipids and proteins is the main cause of sensory, functional and nutritional quality deterioration in meat [3–6] and consumption of such products could have detrimental effect on human health [7].

The major factor controlling the oxidative stability of lipids and proteins is the antioxidant-pro-oxidant balance in the muscle [8–10]. Albeit endogenous antioxidant enzymes in muscle are functional in vivo, their ability to provide protection during *postmortem* may be short-lived [4, 10]. The roles of *postmortem* ageing on lipid oxidation and physicochemical properties of beef [11, 12], mutton [13, 14] and goats [9, 15] have been documented. However, unlike beef [3–5] and mutton [14, 16], the impact of *postmortem* ageing on protein degradation and antioxidant enzyme activities are seldom explored in chevon.

Muscle type plays a vital role in ultimate meat quality [1, 17, 18]. Muscle fibre type is responsible for the variation in meat quality within and between muscles [1, 17, 18]. The *gluteus medius* and *infraspinatus* are classified as fast glycolytic and slow oxidative muscle respectively [1, 17, 18]. Comparing the effect of *postmortem* ageing on these muscles could yield useful information as to the cuts of meat that could be best suited to chilled storage for long periods and most likely to have extended shelf-life. In addition, the biochemical and functional characteristics of each muscle type would likely cause different processing features, which would affect optimum utilization of muscle in value-added products [17, 18]. Despite the established differences between red and white muscles, their response to *postmortem* ageing and the relationships among antioxidant systems, oxidative deterioration and physicochemical properties of goat meat are unclear. Thus, the objective of this study was to compare the *postmortem* changes in antioxidant status, lipid oxidation, myofibrillar protein degradation, metmyoglobin reducing activity, fatty acid profile and physicochemical properties of *gluteus medius* and *infraspinatus* muscles in goats.

## Methods

### Animals, slaughtering procedure and muscle sampling

The study was conducted following the guidelines approved by the Universiti Putra Malaysia Institutional Animal Care and Use Committee. Qualified veterinarians who were members of the research team monitored the animals’ health and welfare.

Twenty Boer crossbred bucks weighing 36.9 ± 0.725 kg (mean ± standard deviation), aged 9–10 months old and raised on 70 % forage (oil palm frond) and 30 % commercial concentrate were sourced from a local farm in Selangor, Malaysia. The goats were fasted overnight with *ad libitum* access to water and slaughtered according to the halal procedure [19] followed by evisceration and carcasses dressing. Each carcass was split along the vertebral column into left and right halves. All analyses were conducted on *infraspinatus* muscle (IS) and *gluteus medius* (GM) on the forelimb and hind limb respectively.

Muscle cuts were sampled from the right halve of each carcass on 0, 1, 4 and 7 d *postmortem*. Twenty cuts from each of *gluteus medius* and *infraspinatus* muscle from twenty bucks were evaluated on each ageing period. On day 0, 60 g of each muscle was dissected from the right half of each carcass, trimmed free of external fat and epimyseal connective tissue and divided into three parts. The first part (15 g) was snap frozen and pulverized in liquid nitrogen with porcelain mortar and pestle to produce a homogenous powder and assigned for the determination of glycogen, pH, antioxidants, fatty acids, myoglobin, metmyoglobin, metmyoglobin reducing activity, lipid-protein oxidation, and SDS-PAGE at d 0. The second part (15 g) was vacuum packaged and stored in a chiller at 4 ± 1 °C for the determination of drip loss. The third part (30 g) was assigned for the determination of colour, cooking loss and shear force on d 0. Muscle samples used for the sensory analysis were obtained from the left half of each carcass. The carcasses (*n* = 20) were hung in the chiller at 4 ± 0.5 °C and subsequent sampling was carried out at 1, 4 and 7 d *postmortem*. At the end of each ageing period, muscle cuts (45 g) devoid of external fat and epimyseal connective tissue were dissected from the carcasses, and divided into two parts. The first part (15 g) was pulverized in liquid nitrogen and assigned as described earlier. The second part (30 g) was assigned for the determination of colour, cooking loss and shear force.

### Determination of muscle glycogen

The glycogen content of pulverized muscles (GM, *n* = 20; IS, *n* = 20 for each ageing period) was determined using EnzyChrom^TM^ Glycogen Assay kit (Cat# E2GN-100, BioAssays, USA) following the procedure of the manufacturer. The assay was based on the hydrolysis of the glycogen content in each sample (150 mg) to glucose by glucoamylase enzyme, oxidized to produce a product, which reacted with OxiRed probe. The colour generated from this reaction was measured at 570 nm using an auto microplate reader (infinite M200, Tecan, Austria). The colour intensity of the reaction product at 570 nm is directly proportional to the glycogen concentration in the sample.

### Determination of muscle pH

The pH of pulverized muscles (GM, *n* = 20; IS, *n* = 20 at each ageing period) was determined following the procedure of AMSA [20] using a pre calibrated portable pH meter (Mettler Toledo, AG 8603, Switzerland). The pH meter was calibrated with a pH 4.0 buffer and then with a pH 7.0 buffer prior to use. Each pulverized sample (0.5 g) was homogenized for 30 s with 10 mL of a 5 mM sodium iodoacetate, 150 mM KCl solution [20]. The pH of the resultant homogenate was measured at 20 ± 1 °C with the aid of the electrode attached to the pH meter.

### Determination of meat colour

Meat colour coordinates were determined according to the method of AMSA [20] using a Colour Flex spectrophotometer (Hunter Lab, Reston, VA, USA) based on the International Commission on Illumination (CIE) Lab-values also known as lightness (L*), redness (a*) and yellowness (b*) with D_65_ illuminant and 10° standard observer, tristimulus values (X,Y,Z) and reflectance at specific wavelength (400–700) nm. The opening aperture size was 1.25 in. and the angle of geometry was 45°/0°. The device was calibrated against black and white reference tiles prior to use. The muscle samples (GM, *n* = 20; IS, *n* = 20) obtained on 0, 1, 4 and 7 d were bloomed for 30 min at 27 °C and the bloomed surface were placed facing the base of the colour flex cup and read. For each sample, triplicate readings for L*, a* and b* values were recorded and then averaged.

### Determination of myoglobin concentration

The extraction and quantification of myoglobin followed the method of Warriss [21]. Three gram of pulverized muscle sample (GM, *n* = 20; IS, *n* = 20 at each ageing period) was homogenized with 15 mL 40 mM potassium phosphate buffer (pH 6.8). The homogenate was incubated on ice for 1 h and centrifuged (Avanti® J-26XPI, BECKMAN COULTER®, USA) at 15, 000 g for 30 min at 4 °C. The supernatant (2 mL) was filtered through Whatman No 1 filter paper and 1.25 mL 40 mM phosphate buffer was added. The absorbance of the mixture was read at 540 nm using a spectrophotometer (Secomam, Domont, France). The concentration of the extracted pigment was estimated using absorbance value, dilution factor, molecular weight of myoglobin (16, 800), and a molar extinction coefficient of 114,000 M^−1^ cm^−1^.

### Determination of metmyoglobin

The percentage Metmyoglobin (%MetMb) was determined as described by Krzywicki [22]. Three gram of pulverized muscle sample (GM, *n* = 20; IS, *n* = 20 at each ageing period) was homogenized with 15 mL ice cold 40 mM phosphate buffer (pH 6.8) for 15 s. The homogenate was incubated on ice for 1 h and centrifuged (Avanti® J-26XPI, BECKMAN COULTER®, USA) at 6000 g for 20 min at 4 °C. The supernatant was filtered through Whatman no 1 filter paper and the absorbance was read at 525, 545 565 and 572 using a spectrophotometer (Secomam, Domont, France). The % MetMb was estimated using the following formula:

$$ \%\mathrm{MetMb} = \left[\hbox{--} 2.514\left(A572/A525\right) + 0.777\left(A565/A525\right) + 0.8\left(A545/A525\right) + 1.098\right] \times 100 $$

### Determination of metmyoglobin reducing activity

Extraction of myoglobin reductase and determination of metmyoglobin reducing activity (MRA) in pulverized meat sample (GM, *n* = 20; IS, *n* = 20 at each ageing period) followed the procedure of Mikkelsen et al. [23].

### Determination of drip loss and cooking loss

Drip and cooking losses were measured according to the method described by Sabow et al. [24] with slight modifications. For drip loss, fresh meat samples (GM, *n* = 20, IS, *n* = 20) at 0 d were individually weighed and recorded as initial weight (W1). The weighed samples were placed into vacuum bags (2.5" x 10.0"; 3.0 mil transparent vacuum pouch with bottom tear notch), labelled, vacuum packaged (98 %) with a chamber Vacuum (CHTC-520LR) and stored at 4 °C. After 1, 4 and 7 d *postmortem*, the samples were removed from the bags, gently blotted dry, weighed and recorded as W_2._ Drip loss was calculated and expressed as the percentage of difference between initial and final weight of sample after storage divide by initial weight of sample as shown in the equation below.

$$ \mathrm{Drip}\;\mathrm{loss}\;\left(\%\right)=\left[\left(\mathrm{W}1\;\hbox{-}\;\mathrm{W}2\right)\div \mathrm{W}1\right]\times 100 $$

For cooking loss, the meat samples (GM, *n* = 20; IS, *n* = 20 at each ageing period) used for colour measurements were individually weighed and recorded as initial weight (W1) and placed in vacuum bags (2.5" x 10.0"; 3.0 mil transparent vacuum pouch with bottom tear notch), labelled, vacuum packaged (98 %) with a chamber Vacuum (CHTC-520LR). The samples were cooked in a pre-heated water bath set at 80 °C. When the internal temperature of the samples reached 80 °C as monitored using a stabbing temperature probe (HI 145–00 thermometer, HANNA® instruments, USA) inserted into the geometric centre of a representative sample, the cooked samples were removed from the water bath, equilibrated to room temperature and removed from the bags, blotted dry without squeezing, and reweighed (W2). The percentage cooking loss was calculated using the following equation:

$$ \mathrm{Cooking}\;\mathrm{loss}\;\left(\%\right)=\left[\left(\mathrm{W}1\;\hbox{-}\;\mathrm{W}2\right)\div \mathrm{W}1\right]\times 100 $$

### Determination of shear force

The samples obtained from the cooking loss analysis were used for the shear force analysis. Meat textural assessment was conducted using the TA.HD plus® texture analyzer (Stable Micro System, Surrey, UK) equipped with a Volodkevitch bite jaw. The equipment was calibrated at 5 kg for weight, 10 mm return distance for height and the blade speed was set at 10 mm/s. The sample preparation was conducted following the procedure of Sazili et al. [25]. From each sample, two replicate blocks (2 cm length × 1 cm width × 1 cm height) were cut parallel to the direction of the muscle fibres and each block was sheared in the centre perpendicular to the longitudinal direction of the fibres. Shear force was reported as the average peak positive force values for all blocks of individual sample.

### Sample preparation for sensory analysis

The samples were trimmed free of external fat and epimyseal connective tissue. The meat samples (20 g each) were placed at the centre of a microwave oven (Elba 20 L, EMO-A2072SV) and microwaved at 450 W for 5 min and the internal temperature was monitored with a stabbing temperature probe (HI 145–00 thermometer, HANNA® instruments, USA). Preliminary investigation conducted to determine the length of cooking time required for the meat samples to reach internal temperature of 80 °C showed that the designated internal temperature could be reached within 5 min at 450 W. No additive was added to the meat samples. Fifteen samples were microwaved at a time. Each sample was cut into a block (2 cm length × 1 cm width × 1.27 cm height), wrapped in aluminium foil, coded with a three-digit random number and kept in the oven at 40 °C until analysis (the holding time was 1 h). A total of 320 samples (GM, *n* = 40; IS, *n* = 40, at each ageing period) were subjected to sensory evaluation.

### Sensory assessors

A consumer type sensory evaluation was conducted as described by Meilgaard et al. [26]. Forty assessors consisting of staff and students of Universiti Putra Malaysia participated in the sensory evaluation. The assessors were solicited via group emails and posters on faculty and departmental notice boards. Assessors were briefed on the sensory protocol and instructed on the parameters to judge [26, 27] using a 9-point hedonic scale. A value of nine indicated like extremely and one indicated dislike extremely. During the briefing section, definition and characteristics of each parameter (tenderness, juiciness, flavour, cooked colour and overall acceptability) and other sensory protocols were explained to the assessors before they entered the booth area. Evaluation was performed in individual booths (temperature, 23 °C; relative humidity, 50 %) under a white fluorescent lighting. Evaluation was conducted for 1 d and two evaluation sessions were held with a 15 min break between sessions. In each session, four different samples (two different muscles at two different periods) were served and sample presentation was randomized. Deionized water was provided to rinse palate after tasting each sample.

### Determination of tocopherol and total carotenoids

Extraction of tocopherol from pulverized muscles (GM, *n* = 20; IS, *n* = 20 at each ageing period) followed the method of Kamal-eldin et al. [28]. Quantification of tocopherol contents was done with Agilent 1200 series HPLC as described by Adeyemi et al. [29]. The carotenoid contents in pulverized meat samples were extracted and quantified as described by Adeyemi et al. [29].

### Antioxidant enzyme activity

The Glutathione peroxidase (GPX) activity in pulverized samples (GM, *n* = 20; IS, *n* = 20 at each ageing period) was measured with the aid of EnzyChrom™ Glutathione Peroxidase Assay Kit EGPX-100, (BioAssay Systems, USA). Superoxide Dismutase (SOD) activity was measured with the aid of Cayman SOD Assay kit (706002, Cayman chemical) while the catalase activity was measured using Cayman Catalase Assay Kit (707002, Cayman chemical) following the manufacturer’s procedure. The amount of sample used for each of SOD, CAT and GPX analysis was 0.5 g.

### Determination of lipid oxidation

Lipid oxidation in pulverized muscle samples (200 mg; GM, *n* = 20; IS, *n* = 20 at each ageing period) was measured as 2-thiobarbituric acid reactive substances (TBARS) using QuantiChrom™ TBARS Assay Kit (DTBA-100, BioAssay Systems, USA) following the manufacturer’s description. The assay was based on the reaction of thiobarbituric acid reactive substance (TBARS) with thiobarbituric acid (TBA) to form a pink coloured product. The colour intensity at 535 nm is directly proportional to TBARS (μM malondialdehyde (MDA) equivalent) concentration in the sample.

### Determination of free thiol content

Protein thiol in pulverized muscles (GM, *n* = 20; IS, *n* = 20 at each ageing period) was quantified according to Elman’s method using 2,2-dithiobis (5-nitropyridine) DTNP [30] with slight modification as described by Morzel et al. [31]. Stock solution containing 4 mg of myofibrillar proteins was dissolved in 3 mL of 100 mM phosphate buffer at pH 8 containing 8 M urea. About 30 μl of 10 mM DTNP (stock solution in ethanol) was added, followed by incubation for 1 h at room temperature. The absorbance at 386 nm was measured using a spectronic®20 GENESYS™ spectrophotometer (Spectronic instruments, USA) against a blank of buffer without protein. The absorbance of the blank was subtracted, and thiol concentration was calculated using an absorption coefficient of 14 mM^−1^ cm^−1^.

### Determination of carbonyl content

The carbonyl content in pulverized muscles (200 mg; GM, *n* = 20; IS, *n* = 20 at each ageing period) was determined using Protein carbonyl colorimetric assay kit (10005020, Cayman Chemical) which utilized 2, 4 dinitrophenylhydrazine (DNPH) reactions to measure carbonyl content in tissues. The amount of protein-hydrozone produced was quantified by a spectrophotometer (infinite M200, Tecan, Austria) at 370 nm and the carbonyl content was standardized to protein concentration.

### Extraction of myofibrillar proteins and determination of protein concentration

Myofibrillar proteins were isolated from pulverized muscles (GM, *n* = 20; IS, *n* = 20 at each ageing period) as described by Morzel et al. [31]. Pulverized muscle (2.5 g) was homogenized for 30 s on ice with 25 mL extraction buffer (25 mM KCl, 150 mM NaCl, 4 mM EDTA, 3 mM MgCl_2_, at pH 6.5) to which protease inhibitor (CALBIOCHEM®, Cat # 55140, EMD Bioscience, Inc. Germany) was added. The homogenate was filtered through 1.0 mm polyethylene strainer to eliminate any remaining collagen. After filtration, the homogenate was incubated at 4 °C with continuous shaking. This was followed by centrifugation at 2000 g for 15 min at 4 °C. The pellet was washed twice with 25 mL of a 50 mM KCl solution at pH 6.4 and once with 25 mL of 20 mM phosphate buffer at pH 6. The pellet was homogenized (T18 digital ULTRA-TURRAX® - IKA, Germany) in 5 mL of 20 mM phosphate buffer for 1 min on ice. The homogenate was centrifuged (Avanti® J-26XPI, BECKMAN COULTER®, USA) at 15,000 g for 20 min at 4 °C. The total protein concentration of an aliquot of the clear supernatant was determined by the method of Bradford [32] using Protein Assay Kit II 500–0002 (Bio-Rad, USA). The protein standards were prepared with Bovine serum albumen [33].

### Sodium dodecyl sulphate polyacrylamide gel electrophoresis (SDS-PAGE)

The extracted myofibrillar proteins were incubated for 10 min at 90 °C in a sample buffer containing 2.3 % (w/v) SDS, 62.5 mM Tris–HCl (pH 6.8), 5 % (v/v) mercaptoethanol, 0.05 % (w/v) bromophenol blue and 30 % (v:v) glycerol. One dimensional SDS-PAGE was performed according to the method of Laemmli [34] using polyacrylamide gels of 8 cm × 5.5 cm (length × width) and 0.8 mm thickness. The resolving gels were over-layered with 4 % stacking gel solution. Samples (30 μg protein) were separated in running buffer (0.025 mol/L Tris base, 0.192 mol/L glycine, 0.1 SDS, pH 8.3) using a mini PROTEAN® Tetra system (Bio-Rad) set at a constant voltage of 120 V and 0.4 A for 90 min. Coomassie blue stain was used to stain the gels. The bands of actin and myosin heavy chain were visualized using GS-800 Calibrated Imaging Densitometer (Bio-Rad, USA).

### Western blotting

The fractionated proteins that were initially separated from the samples based on their molecular weight through gel electrophoresis were transferred from the gel onto polyvinylidene difluoride (PVDF) membranes using Trans-Blot® SD semi-dry transfer system cell (Bio-Rad, USA). Myosin heavy chain was transferred at constant amperage of 250 mA per gel, voltage limit of 25 V for 135 min whereas actin was transferred at the same amperage and voltage for 45 min. After transfer, membranes were blocked for 3 h at room temperature in blocking solution [5 % bovine serum albumin (BSA) in TBS-T buffer (100 mM Tris–HCl; 150 mM NaCl; 0.05 % Tween 20)]. Blots were washed thrice (10 min per wash) in PBS-T and incubated overnight at room temperature with the primary antibody which was diluted 1: 500 in TBST containing 3 % BSA. Monoclonal Anti-Myosin (Skeletal, Fast, produced in mouse; Cat # M4276), Monoclonal Anti-Myosin (Skeletal, Slow, produced in mouse; Cat # M842) and monoclonal Anti actin (produced in rabbit; Cat # A2066 227) from Sigma- Aldrich, USA were the primary antibodies used for myosin heavy chain fast, myosin heavy chain slow and actin respectively. Subsequently, the membranes were incubated with secondary antibody [anti- mouse IgG (whole molecule) - peroxidase, antibody developed in rabbit; (Cat # A9044) from Sigma- Aldrich, USA] diluted 1:10000 in 3 % BSA in TBS-T buffer for 90 min at room temperature. This was followed by washing with TBS-T buffer thrice. The blocked membranes were detected using a DAB substrate kit Code: E885 (DAB SUBSTRATE SYSTEM (aMReSCO®, Solon, DH, USA). Myosin heavy chain and actin band intensities were measured using GS-800 Calibrated Imaging Densitometer (Bio-Rad, USA) using Quantify One® software.

### Fatty acid analysis

The total lipids in pulverized muscle samples (GM, *n* = 20; IS, *n* = 20, at each *postmortem* period) was extracted in chloroform: methanol (2:1, v/v) mixture following the method of Folch et al. [35] modified by Rajion et al. [36]. The extracted fat was transmethylated to fatty acid methyl esters (FAME) using 2 mL 14 % BF_3_ and 2 mL 0.66 N KOH in methanol (Sigma Chemical Co., St. Louis, MO, USA) according to the AOAC [37] method. The standard, column and the gas chromatography settings were described by Adeyemi et al. [38].

### Statistical analysis

The sensory scores were checked for normality using the PROC UNIVARIATE procedure of SAS [39] and were found to be normally distributed. Data obtained from all parameters were analysed using the PROC MIXED procedure of SAS [39] in which muscle, *postmortem* chill storage and interaction between muscle and *postmortem* chill storage were fitted as fixed effects in a repeated measure analysis of variance. Before that, compound symmetry covariance structure, linear and quadratic contrasts were tested in regression analysis and found to have insignificant effects. Means were separated by Tukey HSD test at a significance level of *P* < 0.05. Results were presented as mean ± Standard error.

## Results and discussion

### Muscle pH and glycogen

The physicochemical properties of GM and IS muscles subjected to a 7 d *postmortem* chill storage are shown in Table 1. Regardless of the muscle, the pH and muscle glycogen observed on d 0 were higher (*P* < 0.05) compared with those observed on other storage days. This observation could be due to *postmortem* glycolysis. The cessation of blood circulation at death shifts muscle metabolism from aerobic to anaerobic, which necessitates the conversion of glycogen to lactic acid responsible for the decrease in muscle pH [1, 40]. The pH and glycogen content observed on d 1, 4 and 7 were not significantly different (*P* > 0.05). This indicates that *postmortem* glycolysis was completed during the first 24 h *postmortem*. The current finding is akin to that of Kadim et al. [41] who reported that the pH *of longissimus dorsi, biceps femoris, semitendinosus*, and *semimembranosus* muscles from different Omani breeds of goats observed on d 1 was similar to those observed on d 6 *postmortem*.

**Table 1:** Physicochemical properties of *infraspinatus* (IS) and *gluteus medius* (GM) muscles in goats during *postmortem* chill storage

		Storage time (days)				*P* value	
Parameter	Muscle	0	1	4	7	Storage	Muscle × storage
pH	IS	6.61 ± 0.110 ^ay^	5.87 ± 0.111^by^	5.89 ± 0.140^by^	5.88 ± 0.102^by^	<.0001	
	GM	6.34 ± 0.031^ax^	5.60 ± 0.101^bx^	5.60 ± 0.310^bx^	5.59 ± 0.221^bx^	0.001	0.091
	*P* value	0.012	0.001	0.012	0.021		
Glycogen (mg/g)	IS	1.35 ± 0.010^a^	0.68 ± 0.023^by^	0.64 ± 0.011^by^	0.65 ± 0.010 ^by^	<.0001	
	GM	1.30 ± 0.030^a^	0.58 ± 0.012^bx^	0.56 ± 0.011 ^bx^	0.57 ± 0.012 ^bx^	<.0001	0.100
	*P* value	0.213	0.021	0.011	0.019		
Drip loss (%)	IS	-	3.55 ± 0.342^c^	4.67 ± 0.481^b^	6.05 ± 0.731^ay^	<.0001	
	GM	-	2.80 ± 0.110^c^	3.98 ± 0.210^b^	4.72 ± 0.140^ax^	<.0001	0.104
	*P* value		0.090	0.080	0.023		
Cooking loss (%)	IS	38.22 ± 0.532^ax^	33.27 ± 0.230^bx^	34.54 ± 0.682^b^	34.48 ± 0.920^b^	<.0001	
	GM	42.12 ± 0.651^ay^	37.87 ± 0.471^by^	36.67 ± 0.701^b^	36.79 ± 0.991^b^	0.0431	0.120
	*P* value	0.029	0.014	0.072	0.064		
Shear force (kg)	IS	1.21 ± 0.011^ax^	0.89 ± 0.042^bx^	0.83 ± 0.011^bcx^	0.77 ± 0.020^c^	0.0275	0.224
	GM	1.32 ± 0.041^ay^	0.94 ± 0.021^by^	0.88 ± 0.012^bcy^	0.80 ± 0.042^c^	<.0001	
	*P* value	0.023	0.015	0.016	0.201		
Lightness (L*)	IS	32.64 ± 0.470^b^	33.26 ± 0.850^b^	37.77 ± 1.152^a^	37.52 ± 0.464^a^	0.004	0.126
	GM	34.04 ± 0.220^b^	34.98 ± 0.111^b^	37.22 ± 1.201^a^	38.02 ± 1.013^a^	0.002	
	*P* value	0.061	0.258	0.078	0.069		
Redness (a*)	IS	13.24 ± 0.221^ay^	14.12 ± 0.261^ay^	10.70 ± 0.690^b^	8.83 ± 0.180^cx^	<.0001	0.113
	GM	11. 20 ± 0.142^ax^	12.02 ± 0.111^ax^	10.23 ± 0.160^b^	9.45 ± 0.171^cy^	0.002	
	*P* value	0.012	0.038	0.500	0.019		
Yellowness (b*)	IS	13.38 ± 0.470	13.76 ± 0.150	11.64 ± 1.442	13.92 ± 0.882	0.288	0.183
	GM	13.14 ± 0.231	12.98 ± 0.712	12.47 ± 0.221	12.33 ± 0.332	0.543	
	*P* value	0.231	0.106	0.114	0.321		
Myoglobin (mg/g)	IS	3.21 ± 0.143^ay^	2.94 ± 0.072^ay^	2.74 ± 0.083^b^	2.68 ± 0.111^b^	0.020	0.156
	GM	2.89 ± 0.110^ax^	2.80 ± 0.122^ax^	2.71 ± 0.041^b^	2.60 ± 0.122^b^	0.013	
	*P* value	0.032	0.022	0.091	0.213		
Metmyoglobin (%)	IS	2.83 ± 0.252^d^	7.19 ± 0.391^c^	12.77 ± 0.480^b^	20.47 ± 0.571^ay^	<.0001	0.120
	GM	2.91 ± 0.433^d^	7.39 ± 0.222^c^	12.35 ± 0.232^b^	18.99 ± 0.110^ax^	0.002	
	*P* value	0.156	0.412	0.210	0.046		
MRA^1^ (nmol/min/g)	IS	200.06 ± 2.081^a^	189.50 ± 5.001^ab^	180.44 ± 6.562^b^	169.08 ± 4.920^c^	0.043	0.189
	GM	212.77 ± 3.112^a^	200.45 ± 3.232^b^	190.22 ± 4.113^c^	151.90 ± 3.120^d^	0.004	
	*P* value	0.216	0.120	0.147	0.548		

^a, b c^ means having different superscripts along the same row are significantly different (*P* < 0.05). ^x, y^ means having different superscripts along the same column are significantly different (*P* < 0.05). ^1^metmyoglobin reducing activity

The IS muscle had greater (*P* < 0.05) glycogen content than GM on d 1, 4 and 7. In addition, the IS had higher (*P* < 0.05) pH than GM throughout storage. This finding could be attributed to the inherent differences in fibre types and *postmortem* energy metabolism between the muscles [17, 18]. Glycolytic muscles are more responsive to *postmortem* glycolysis because they have higher concentration of inorganic phosphate, which stimulates the conversion of glycogen to lactic acid [1, 17]. The current observations are in tandem with those of Sitthigripong et al. [42] who observed a higher pH in *supraspinatus*, *infraspinatus* and *psoas major* compared with *longissimus dorsi* from Boer crossbred goats. There was no significant interaction between muscle type and *postmortem* chill storage for the pH and glycogen content in goat meat.

### Drip and cooking losses

The drip and cooking losses of IS and GM muscles during *postmortem* refrigerated storage are shown in Table 1. Regardless of muscle, drip loss increased (*P* < 0.05) over storage. This observation could be due to the reduction in the available space (steric effects) for water to reside in the muscle due to the formation of cross-bridges between the thick and thin myofibrillar filaments during rigor development [43, 44]. In addition, as the muscle reaches rigor, the pH of the tissue nears the isoelectric point of many of the major proteins (particularly myosin), thereby reducing the amount of water that is attracted to protein structures in the myofibril [45]. Similar increase in drip loss was observed during *postmortem* ageing of chevon [9, 46].

The IS muscle had higher (*P* < 0.05) drip loss than GM on d 7 *postmortem*. This observation was contrary to our expectation given the higher pH in IS compared with GM. A high ultimate pH, above the isoelectric point of myofibrillar proteins enhances the water holding capacity of meat [43, 45]. Thus, the higher drip loss in IS could be due to its greater (*P* < 0.05) degradation of myofibrillar proteins (Table 4) and greater carbonyl content on d 7 (Table 3). There was a positive correlation between drip loss and degradation of myofibrillar proteins during ageing of meat [45, 47].

Irrespective of muscle, the cooking loss observed on d 0 was greater (*P* < 0.05) than that observed on other storage days. This observation could be due to the degradation of cytoskeletal proteins, which reduces rigor-induced lateral shrinkage of myofibrils thereby reducing the amount of water expelled [48]. This observation corroborates the findings of Sabow et al. [24] who observed that d 0 cooking loss in *longissimus lumborum* muscle in goats was greater than those observed on other storage days.

Cooking loss was stable from 1 to 7 d *postmortem* in both muscles. This observation could be due to the stability of pH. The GM had higher (*P* < 0.05) cooking loss than IS on d 0 and 1 *postmortem*. This could be attributed to the higher (*P* < 0.05) pH of IS compared with GM. The induction of a high ultimate pH in muscle will diminish that particular portion of the cooking loss, which is due to the exudation of moisture [1, 49]. In addition, the higher cooking loss in GM could be due to its lower intramuscular fat than IS (Table 5). Intramuscular fat possibly loosen up the microstructure, thus allowing more water to be entrained [1, 45]. Similarly, glycolytic *semitendinosus* muscle had higher cooking loss than oxidative *supraspinatus* muscle in goats [50]. There was no significant interaction between muscle type and chill storage for drip and cooking losses in goat meat.

### Shear force values

*Postmortem* chill storage was a significant (*P* < 0.05) source of variation affecting shear force in goat meat (Table 1). Regardless of the muscle, there was a reduction (*P* < 0.05) in shear force from d 0 to 7 *postmortem. Postmortem* improvement in meat tenderness could be due to the weakening of myofibrillar structure by endogenous muscle proteinases [1, 40]. Similarly, a decrease in shear force was observed during *postmortem* ageing of chevon [9, 24]. The IS had lower (*P* < 0.05) shear force than GM on d 0, 1 and 4 *postmortem*. This observation could be due to the higher (*P* < 0.05) intramuscular fat (Table 5) and lower cooking loss (Table 1) in IS compared with GM. Intramuscular fat increases expansion of fat cell in the perimysial connective tissue, which forces muscle bundles apart, thus opening up the muscle structure [1]. This finding could also be due to the high ultimate pH in IS which favours calpain activities thereby improving meat tenderness [51]. The relative proportions of connective tissue vary between muscles and, in part, account for the relative toughness of meat [1]. White muscles have a higher connective tissue than red muscles [1]. Terrescano et al. [52] observed that oxidative *psoas major* and *diaphragma* had lower shear force, total collagen and insoluble collagen compared with glycolytic *gluteus medius* and *semimembranosus* in beef. In addition, Aghwan [50] observed a lower shear force in oxidative *supraspinatus* muscle than glycolytic *semitendinosus* muscles in goats.

### Meat colour

*Postmortem* chill storage influenced (*P* < 0.05) the redness (a*) and lightness (L*) of GM and IS muscles in goats (Table 1). Neither muscle nor chill storage influenced yellowness (b*) of goat meat. The L* and a* values increased and decreased (*P* < 0.05) respectively from d 1 to 7 *postmortem*. This observation could be due to the decrease (*P* < 0.05) in myoglobin concentration and metmyoglobin reducing activity (MRA) and the increase (*P* < 0.05) in % metmyoglobin as storage progressed. The improvement in L* and the decrease in a* over storage concur with previous findings in chevon [9], beef [12] and mutton [13]. The IS had higher redness compared with GM on day 0 and 1 *postmortem*. This could be due to the higher concentration of oxygenated myoglobin in IS compared with GM [1, 17, 18]. Similarly, Mercier et al. [53] reported a higher a* in oxidative *sartorius* than glycolytic *pectoralis major* in turkey.

### Myoglobin (Mb), % metmyoglobin (MetMb) and metmyoglobin reducing activity (MRA)

*Postmortem* chill storage had significant (*P* < 0.05) effect on Mb, and % MetMb in IS and GM muscles in goats (Table 1). Irrespective of muscle, the concentration of Mb observed on d 0 and 1 did not differ (*P* > 0.05) but was greater (*P* < 0.05) than those observed on d 4 and 7. The % MetMb increased (*P* < 0.05) over storage in both muscles. These observations could be due to the oxidation of Mb or oxymyoglobin to form MetMb [12, 13]. Similarly, the % MetMb of beef patties subjected to an 8 d [12] and a 10 d [11] chill storage increased as storage continued.

The IS had higher Mb than GM on d 0 and 1. In addition, the % MetMb was higher in IS compared with GM on d 4 and 7 *postmortem*. These observations lend credence to the higher (*P* < 0.05) redness on d 0 and 1 and lower redness on d 7 in IS compared with GM. These observations are consistent with those of Renerre et al. [4] who observed that oxidative *diaphragma* and *psoas major* had greater % MetMb and lower colour stability than glycolytic *longissimus lumborum* and *tensor fasciae latae* during an 8 d *postmortem* storage of beef.

The MRA decreased (*P* < 0.05) as *postmortem* conditioning progressed (Table 1). On d 7, there was a 15 % and 23 % reduction in the original MRA in the GM and IS respectively. This finding is in tandem with that of Madhavi and Carpenter [54] who observed a 20 % reduction in MRA of beef from d 2 to 21 *postmortem*. In contrast, there was a significant increase in the MRA of beef patties over an 8 d [12] and a 10 d [11] refrigerated storage. Bekhit et al. [13] observed that the MRA in ovine *longissimus* muscle was stable throughout a 10 d chill storage. The discrepancies among studies with respect to the effect of *postmortem* storage on MRA of meat could be due to the differences in methodology employed, the rate of myoglobin oxidation and efficacy of one or more enzymes required for reducing the formed metmyoglobin [55]. The MRA was not significantly different (*P* > 0.05) between GM and IS throughout storage. Interaction between muscle type and chill storage was not significant (*P* > 0.05) for myoglobin, % metmyoglobin and MRA in goat meat.

### Sensory attributes

The effects of muscle type and *postmortem* chill storage on meat sensory scores are presented in Table 2. In both muscles, assessors ranked meat samples tasted on d 1, 4 and 7 as more tender (*P* < 0.05) and having greater overall acceptance (*P* < 0.05) than d 0 samples. This could be due to the *postmortem* weakening of myofibrillar proteins in the course of ageing [1, 51]. This coincides with the decrease (*P* < 0.05) in the instrumental shear force as storage progressed (Table 1). Consumers ranked d 0 meat samples as juicer (*P* < 0.05) compared with those tasted on other storage days. Chill storage had no effect (*P* > 0.05) on the liking of flavour and cooked colour of goat meat. Muscle type had no effect (*P* > 0.05) on the consumer preference for cooked colour, flavour, juiciness and overall acceptability of goat meat throughout storage. However, consumers ranked IS as tenderer (*P* < 0.05) than GM on d 1, 4 and 7 *postmortem*. This could be attributed to the lower (*P* < 0.05) cooking loss and instrumental shear force in IS compared with GM (Table 1). A lower cooking loss is likely to improve tenderness because a given cross-sectional area of meat sample will contain more water and less structural components [49]. There was no significant interaction (*P* > 0.05) between muscle type and chill storage for the sensory scores of goat meat.

**Table 2:** Sensory attributes of *infraspinatus* (IS) and *gluteus medius* (GM) muscles in goats during *postmortem* chill storage

		Storage time (days)				*P* value	
Parameter	Muscle	0	1	4	7	Storage	Muscle × storage
Juiciness	IS	7.81 ± 0.200^a^	6.84 ± 0.161^b^	6.19 ± 0.140^b^	6.98 ± 0.101^b^	0.043	
	GM	7.73 ± 0.030^a^	6.70 ± 0.322^b^	6.10 ± 0.210^b^	6.89 ± 0.520^b^	0.023	0.091
	*P* value	0.101	0.201	0.463	0.110		
Tenderness	IS	6.35 ± 0.010^a^	8.38 ± 0.021^bx^	8.64 ± 0.010^bx^	8.60^by^ ± 0.012	0.041	
	GM	6.30 ± 0.031^a^	7.78 ± 0.010^by^	7.65 ± 0.010^by^	7.99^bx^ ± 0.011	0.011	0.108
	*P* value	0.213	0.011	0.019	0.009		
Flavor	IS	7.70 ± 0.441	7.12 ± 0.433	7.43 ± 0.842	7.31 ± 0.774	0.876	
	GM	7.65 ± 0.532	7.19 ± 0.192	7.19 ± 0.141	7.60 ± 0.671	0.231	0.428
	*P* value	0.890	0.090	0.080	0.083		
Cooked colour	IS	7.22 ± 0.531	7.27 ± 0.231	7.54 ± 0.682	7.80 ± 0.922	0.331	
	GM	7.12 ± 0.652	7.87 ± 0.472	7.17 ± 0.701	7.79 ± 0.991	0.234	0.101
	*P* value	0.392	0.104	0.172	0.106		
Overall acceptability	IS	7.51 ± 0.712^a^	8.33 ± 0.760^b^	8.03 ± 0.820^b^	8.03 ± 0.510^b^	0.025	0.224
	GM	7.52 ± 0.541^a^	8.45 ± 0.720^b^	8.05 ± 0.811^b^	8.03 ± 0.842^b^	0.043	
	*P* value	0.135	0.111	0.147	0.126		

^a, b c^ means having different superscripts along the same row are significantly different (*P* < 0.05). ^x, y^ means having different superscripts along the same column are significantly different (*P* < 0.05)

### Antioxidant enzyme activities, tocopherol and carotenoid

The antioxidant enzyme activities and concentration of carotenoid and tocopherol in GM and IS muscles during chill storage are shown in Table 3. Regardless of the muscle, the catalase (CAT), glutathione peroxidase (GPX) and superoxide dismutase (SOD) activities were unaffected (*P* > 0.05) by chill storage. There was no significant correlation (data not shown) between antioxidant enzyme activities and indicators of quality deterioration in goat meat. The effects of chill storage on antioxidant enzyme activities in meat has yielded inconsistent results in the published literature. Renerre et al. [4] observed that the GPX and CAT activities in bovine muscles on d 1 was similar to those observed on d 8 *postmortem*. However, the authors observed that SOD activity declined over the 8 d storage. Renerre et al. [8] observed a non-significant reduction in SOD but a significant decrease in GPX in *pectoralis major* muscle of turkey over a 9 d chill storage.

The activities of SOD and CAT were greater (*P* < 0.05) in IS compared with GM throughout storage. However, GPX activity was not influenced by muscle type. These observations are consistent with those of Renerre et al. [4] who observed higher SOD and CAT activities in oxidative *diaphragma* compared with glycolytic *longissimus lumborum* during an 8 d *postmortem* storage of beef. In turkey, Renerre et al. [8] observed that oxidative *sartorius* had higher SOD and CAT than glycolytic *pectoralis major* during a 9 d *postmortem* ageing. Interaction between muscle type and chill storage was not significant for antioxidant enzyme activities in goat meat. The increase in the activities of CAT and SOD in oxidative muscles could be due to a positive feedback mechanism in response to rising oxidative deteriorations [4, 8].

Muscle type had no effect on the concentration of carotenoid, α, γ and δ-tocopherol. In contrast, levels of α-tocopherol were found to be highest in beef *psoas major* and *gluteus medius* and lowest in *longissimus thoracis* and *longissimus lumborum* while moderate levels of α-tocopherol occurred in *semimembranosus* [56]. There was a decline (*P* < 0.05) in the concentration of carotenoid, α, and γ-tocopherol as storage progressed. This finding is consistent with that of Irie et al. [57] who observed a reduction in the concentration of α-tocopherol in Japanese beef as *postmortem* storage progressed. There was no significant interaction between muscle type and *postmortem* ageing for the concentration of tocopherols and total carotenoids in goat meat. The concentration of tocopherols and total carotenoids was correlated (data not shown) with indicators of quality deterioration in goat meat. This suggests that the tocopherol and carotenoids may be the primary driver for *postmortem* oxidative stability of goat meat.

### Lipid oxidation

The effect of *postmortem* storage on lipid oxidation measured as thiobarbituric acid reactive substance (TBARS) is shown in Table 3. There was an increase (*P* < 0.05) in TBARS values in both muscles as *postmortem* storage progressed. This observation is consistent with findings in broiler meat [58, 59], beef [4, 12] and chevon [9, 15]. Despite the increase in TBARS over storage, the range of TBARS values observed in the current study was below the threshold (0.6 mg MDA/kg) specified for abnormal flavour development in meat [60].

**Table 3:** Antioxidant status, protein degradation and lipid oxidation in *infraspinatus* (IS) and *gluteus medius* (GM) muscles in goats during *postmortem* chill storage

		Storage time (days)				*P* value	
Parameter	Muscle	0	1	4	7	Storage	Storage × muscle
α-tocopherol (mg/kg)	IS	2.19 ± 0.130^a^	2.01 ± 0.100^b^	1.43 ± 0.061^c^	1.16 ± 0.020^c^	<.0001	0.192
	GM	2.14 ± 0.111^a^	1.93 ± 0.120^b^	1.40 ± 0.032^c^	1.23 ± 0.040^c^	0.0012	
	*P* value	0.217	0.140	0.223	0.123		
γ-tocopherol (mg/kg)	IS	0.69 ± 0.051^a^	0.65 ± 0.052^a^	0.41 ± 0.010^b^	0.23 ± 0.020^c^	<.0001	0.237
	GM	0.70 ± 0.022^a^	0.65 ± 0.021^a^	0.44 ± 0.040^b^	0.27 ± 0.010^c^	<.0001	
	*P* value	0.532	0.112	0.267	0.417		
δ-tocopherol (mg/kg)	IS	0.08 ± 0.011	0.08 ± 0.012	0.07 ± 0.011	0.07 ± 0.011	0.109	0.280
	GM	0.12 ± 0.031	0.10 ± 0.011	0.09 ± 0.021	0.09 ± 0.031	0.204	
	*P* value	0.211	0.234	0.091	0.094		
Total carotenoid (mg/kg)	IS	0.27 ± 0.010^a^	0.23 ± 0.011^a^	0.18 ± 0.012^b^	0.11 ± 0.010^c^	<.0001	0.447
	GM	0.25 ± 0.030^a^	0.22 ± 0.012^a^	0.15 ± 0.041^b^	0.10 ± 0.030^c^	0.004	
	*P* value	0.901	0.548	0.071	0.361		
Catalase^1^	IS	1828.9 ± 27.23^y^	1820.0 ± 20.83^y^	1844.2 ± 21.91^y^	1816.3 ± 26.80^y^	0.190	0.135
	GM	1666.2 ± 11.80^x^	1767.0 ± 10.20^x^	1515.2 ± 12.11^x^	1700.2 ± 17.10^x^	0.219	
	*P* value	0.012	0.009	0.034	0.022		
SOD^2^	IS	3.14 ± 0.161^y^	3.07 ± 0.121^y^	3.31 ± 0.212^y^	3.17 ± 0.05^y^	0.742	0.221
	GM	2.70 ± 0.132^x^	2.45 ± 0.143^x^	2.87 ± 0.111^x^	2.78 ± 0.05^x^	0.092	
	*P* value	0.030	0.002	0.043	0.012		
GPX^3^	IS	66.75 ± 2.621	63.42 ± 3.500	65.03 ± 2.772	63.01 ± 0.940	0.806	0.156
	GM	65.20 ± 1.600	59.89 ± 1.250	63.11 ± 2.112	62.22 ± 1.332	0.418	
	*P* value	0.190	0.090	0.301	0.128		
TBARS (mg MDA/kg)	IS	0.12 ± 0.010^c^	0.15 ± 0.011^c^	0.19 ± 0.010^b^	0.28 ± 0.010^a^	<.0001	0.173
	GM	0.11 ± 0.010^c^	0.13 ± 0.020^c^	0.18 ± 0.030^b^	0.26 ± 0.031^a^	<0.001	
	*P* value	0.130	0.273	0.112	0.367		
Carbonyl (nmol/mg protein)	IS	1.08 ± 0.041^a^	1.43 ± 0.130^a^	2.47 ± 0.291^b^	3.63 ± 0.120^cx^	<.0001	0.451
	GM	1.09 ± 0.010^a^	1.35 ± 0.120^a^	2.50 ± 0.122^b^	3.01 ± 0.221^cy^	<.0001	
	*P* value	0.231	0.154	0.347	0.035		
Free thiol (nmol/mg protein)	IS	52.89 ± 1.671^a^	49.75 ± 1.302^a^	45.91 ± 1.380^b^	39.25 ± 1.711^c^	0.014	0.234
	GM	55. 82 ± 1.220^a^	52.86 ± 1.112^a^	48.00 ± 1.091^b^	41.01 ± 1.000^c^	0.021	
	*P* value	0.213	0.437	0.815	0.200		

^a, b c^ means having different superscripts along the same row are significantly different (*P* < 0.05). ^x, y^ means with different superscript along the same column are significantly different (*P* < 0.05). ^1^catalase activity is expressed as nmol.H_2_O_2_/min/mg protein. ^2^superoxide dismutase is expressed as the amount of enzyme needed to inhibit 50 % dismutation of the superoxide radical. ^3^glutathione peroxidase activity is expressed as nmoles NADPH oxidized/min/mg protein

Muscle type had no effect (*P* < 0.05) on TBARS value throughout storage. This could be due to the similar concentration of carotenoid and tocopherol in the muscles. In contrast, Renerre et al. [4] observed that *diaphragma* and *psoas major* had higher TBARS values compared with *longissimus lumborum* and *tensor fasciae latae* during an 8 d *postmortem* storage of beef. In turkey, Renerre et al. [8] observed that *sartorius* muscle had higher TBARS value than *pectoralis major* muscle during a 9 d *postmortem* ageing. The interaction between muscle type and *postmortem* chill storage was not significant for TBARS values in goat meat.

### Free thiol content

The oxidation of accessible free thiol (the sulfhydryl group) from cysteine residues corresponds to the loss of thiols [31]. *Postmortem* chill storage had a significant (*P* < 0.05) effect on the free thiol content in goat meat. The thiol concentration reduced from 52.89 to 39.25 nmol/mg protein in IS which represented a 26 % reduction while in the GM, free thiol reduced from 55.82 to 41.01 nmol/mg protein, which represented a 27 % reduction. Similarly, Petron et al. [16] observed about 23 % loss of thiol groups in mutton from 4 to 8 d *postmortem*. Muscle type had no effect on free thiol content. In contrast, Mercier et al. [53] observed lower free thiol in oxidative *sartorius* than glycolytic *pectoralis major* of turkey meat during a 9 d *postmortem* chill storage. There was no significant interaction between the muscle type and chill storage for thiol concentration in goat meat.

### Carbonyl content

The formation of carbonyl is one of the most significant chemical modifications of oxidized proteins [4, 14, 16]. The carbonyl content of goat meat increased (*P* < 0.05) over storage. This observation is akin to findings in beef [4] turkey [8] and lamb [14, 16]. The IS had higher (*P* < 0.05) carbonyl content than GM on d 7 *postmortem*. Similarly, Renerre et al. [8] observed a higher carbonyl content in oxidative *sartorius* muscle than glycolytic *pectoralis major* muscle during a 9 d *postmortem* chill storage of turkey. In beef, Renerre et al. [4] observed greater carbonyl content in *diaphragma* muscle than *longissimus dorsi* muscle. In contrast, Martinaud et al. [61] observed a higher carbonyl content in *longissimus lumborum* compared with *diaphragma pedialis* of beef during a 10 d chill storage. There was no significant interaction between the muscle type and chill storage for carbonyl concentration in goat meat.

### Distribution of myofibrillar proteins

**Fig. 1:**
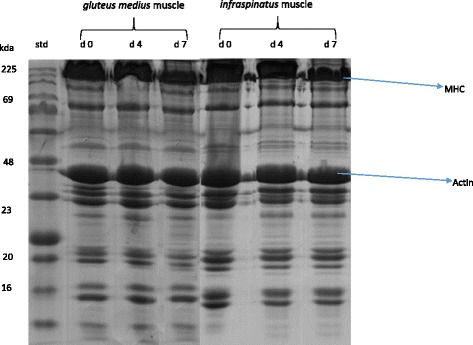
SDS-PAGE of myofibrillar proteins of *gluteus medius* (GM) and *infraspinatus* (IS) muscles of goats at 0, 4 and 7 d *postmortem*. Std = standard. MHC = myosin heavy chain

**Fig. 2:**
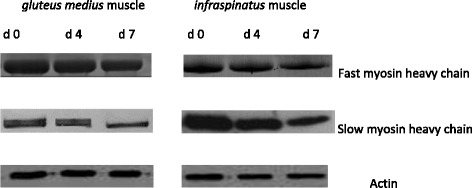
A representative Western- Blots of slow myosin heavy chain, fast myosin heavy chain and actin in *infraspinatus* and *gluteus medius* muscle in goats during *postmortem* chill storage

A representative gel showing the SDS-PAGE of myofibrillar protein pattern in IS and GM muscles in goats during chill storage is shown in Fig. 1. A representative Western-Blots of slow MHC, fast MHC and actin in IS and GM muscles in goats during chill storage is shown in Fig. 2. Table 4 shows the reflective density of myofibrillar proteins in IS and GM muscles during *postmortem* chill storage.

**Table 4:** Reflective density of myofibrillar proteins in *infraspinatus* (IS) and *gluteus medius* (GM) muscles in goats during *postmortem* chill storage

		Storage time (days)			*P* value	
Parameter	Muscle	0	4	7	Storage	Storage × muscle
Fast MHC^1^ (density/mm^2^)	IS	26.54 ± 0.120^ax^	23.00 ± 0.209^bx^	20.49 ± 0.231^cx^	0.004	0.091
	GM	94.33 ± 0.100^ay^	88.44 ± 0.707^by^	81.20 ± 0.309^cy^	0.023	
	*P* value	0.001	0.002	0.001		
% loss of fast MHC	IS		13.33 ± 0.900^by^	22.61 ± 2.110^ay^	0.009	0.09
	GM		6.24 ± 0.001^bx^	13.91 ± 0.148^ax^	0.012	
	*P* value		0.021	0.033		
Slow MHC^1^ (density/mm^2^)	IS	100.01 ± 4.011^ay^	85.00 ± 2.991^by^	63.44 ± 3.311^cy^	0.001	0.102
	GM	24.10 ± 0.122^ax^	20.30 ± 2.00^bx^	17.00 ± 2.121^cx^	0.001	
	*P* value	0.009	0.011	0.001		
% loss of slow MHC	IS		15.00 ± 0.020^b^	36.56 ± 2.067^ay^	0.032	0.110
	GM		15.76 ± 0.341^b^	29.16 ± 2.019^ax^	0.016	
	*P* value		0.130	0.002		
Actin (density/mm^2^)	IS	13.76 ± 0.400^x^	13.50 ± 1.207^x^	13.40 ± 0.610^x^	0.100	0.102
	GM	21.00 ± 0.211^y^	20.69 ± 0.144^y^	20.50 ± 0.201^y^	0.120	
	*P* value	0.009	0.021	0.018		
% loss of actin	IS		1.16 ± 0.001	2.61 ± 0.863	0.091	0.087
	GM		1.47 ± 0.001	2.38 ± 0.152	0.070	
	*P* value		0.114	0.185		

^a, b c^ means having different superscripts along the same row are significantly different (*P* < 0.05). ^x, y^ means with different superscript along the same column are significantly different (*P* < 0.05). ^1^myosin heavy chain

The Western-blot analysis and the reflective band intensity showed that the IS had greater (*P* < 0.05) reflective density of slow MHC than GM throughout storage. In contrast, the western blots and reflective density of fast MHC and actin were greater (*P* < 0.05) in GM compared with IS throughout storage. This finding was expected and it relates to the differences in the biochemical and functional properties of muscle fibres in the muscles. The greater fast MHC and actin in GM muscle reflects its function as a white/glycolytic muscle used for short and rapid contractions [1, 17, 18]. The greater slow MHC in IS indicates its function as a red/oxidative muscle used to carry out slow and sustained contraction for prolonged period without fatigue [1, 17, 18]. The current distribution pattern of myofibrillar protein is consistent with those of Sazili et al. [25] who found that the level of slow MHC in *supraspinatus* was significantly greater than that of *longissimus lumborum* and *semitendinosus* muscles in sheep. In addition, the fast MHC content in *longissimus lumborum* and *semitendinosus* muscles was greater than that of *supraspinatus* muscle in sheep [25]. *Postmortem* chill storage had no effect (*P* > 0.05) on the distribution of myofibrillar proteins in goat meat.

### Degradation of myofibrillar proteins

Chill storage had a significant effect (*P* < 0.05) on the SDS-PAGE patterns, western blots, reflective density and % loss of fast MHC and slow MHC in goat meat. Regardless of muscle, the western blots and the reflective density of slow MHC and fast MHC decreased (*P* < 0.05) over storage. The degradation of MHC over storage is consistent with findings in camel meat [62], chevon [63] and rabbit meat [64]. In contrast, Bandman and Zdanis [65] posited that myosin was resilient after 28 d *postmortem* ageing of beef at 4 °C. In addition, the MHC in rabbit meat did not degrade during a 7 d *postmortem* ageing [66].

The percentage loss (degradation) of fast MHC was greater (*P* < 0.05) in IS than GM on d 4 and 7 *postmortem*. Similarly, the degradation of slow MHC was greater (*P* < 0.05) in IS than GM on d 7 *postmortem*. This observation is consistent with the greater carbonyl content in IS compared with GM on day 7 *postmortem*. Oxidative muscles contain greater concentration of free iron than glycolytic muscles, and the free iron could catalyse the oxidative degradation of proteins [1, 4, 8].

*Postmortem* ageing did not affect (*P* > 0.05) the reflective density of actin in IS and GM muscles in goats. Similarly, actin underwent little or no degradation when rabbit meat [64, 66] and chevon [64] were subjected to chill storage. In contrast, actin was degraded during a 9 d chill storage of camel meat [62].

### Intramuscular fat and fatty acid composition

The fatty acid (FA) composition and intramuscular fat (IMF) of IS and GM muscles subjected to *postmortem* chill storage is shown in Table 5. The IS muscle had higher (*P* < 0.05) IMF compared with GM. High IMF is a known characteristic of oxidative muscles which suggests the need to meet the high energy demand for cellular metabolisms by fat oxidation [67, 68]. Similarly, oxidative *triceps brachiii* had greater intramuscular fat than glycolytic *gluteus medius* in grass-fed beef [68].

**Table 5:** Intramuscular fat (mg/100 g) and fatty acid composition (% of total fatty acid) of *infraspinatus* (IS) and *gluteus medius* (GM) muscles in goats during *postmortem* chill storage

		Storage time (days)			*P* value	
Parameter	Muscle	0	4	7	Storage	Muscle × storage
C12:0	IS	0.26 ± 0.010^c^	0.30 ± 0.012^b^	0.34 ± 0.010^a^	<.0001	0.219
	GM	0.30 ± 0.011^b^	0.32 ± 0.021^b^	0.35 ± 0.021^a^	0.013	
	*P* value	0.122	0.221	0.191		
C14:0	IS	1.89 ± 0.021^c^	2.00 ± 0.010^b^	2.14 ± 0.020^a^	0.001	0.130
	GM	1.98 ± 0.010^b^	2.12 ± 0.010^a^	2.14 ± 0.010^a^	0.011	
	*P* value	0.110	0.210	0.923		
C14:1	IS	0.10 ± 0.010	0.09 ± 0.011	0.10 ± 0.010	0.429	0.213
	GM	0.12 ± 0.121	0.11 ± 0.120	0.12 ± 0.110	0.500	
	*P* value	0.712	0.115	0.345		
C15:0	IS	0.54 ± 0.021^c^	0.65 ± 0.020^b^	0.80 ± 0.040^a^	<.0001	0.143
	GM	0.60 ± 0.011^b^	0.64 ± 0.031^b^	0.75 ± 0.011^a^	0.014	
	*P* value	0.201	0.345	0.167		
C15:1	IS	0.60 ± 0.009^a^	0.60 ± 0.012^a^	0.70 ± 0.010^b^	0.041	
	GM	0.65 ± 0.008^a^	0.67 ± 0.012^a^	0.72 ± 0.011^b^	0.020	0.230
	*P* value	0.144	0.125	0.223		
C16:0	IS	19.20 ± 0.210^c^	20.97 ± 0.310^b^	21.22 ± 0.667^a^	<.0001	0.331
	GM	19.41 ± 0.112^c^	20.44 ± 0.121^b^	21.10 ± 0.325^a^	0.003	
	*P* value	0.221	0.298	0.145		
C16:1n-7	IS	1.20 ± 0.021^b^	1.79 ± 0.061^a^	1.75 ± 0.027^a^	<.0001	
	GM	1.98 ± 0.010^b^	2.30 ± 0.020^a^	2.33 ± 0.089^a^	0.042	0.219
	*P* value	0.191	0.063	0.061		
C17:0	IS	0.62 ± 0.036^b^	0.64 ± 0.020^b^	0.74 ± 0.021^a^	0.017	
	GM	0.71 ± 0.017^b^	0.74 ± 0.010^b^	0.80 ± 0.041^a^	0.042	0.117
	*P* value	0.129	0.212	0.156		
C17:1	IS	0.41 ± 0.010^b^	0.65 ± 0.023^a^	0.70 ± 0.021^a^	<.0001	
	GM	0.43 ± 0.011^c^	0.59 ± 0.012^b^	0.73 ± 0.012^a^	<.0001	0.127
	*P* value	0.110	0.201	0.120		
C18:0	IS	20.21 ± 0.450 ^c^	21.25 ± 0.300 ^b^	22.50 ± 0.561 ^a^	0.031	
	GM	20.41 ± 0.210 ^c^	22.01 ± 0.110 ^b^	23.21 ± 0.472 ^a^	0.042	0.222
	*P* value	0.161	0.540	0.07		
C18:1n-9	IS	27.52 ± 0.705	27.07 ± 0.209	27.09 ± 0.767	0.142	
	GM	26.23 ± 0.338	26.14 ± 0.217	28.24 ± 0.189	0.213	0.223
	*P* value	0.649	0.136	0.598		
C18:1 *t*-11	IS	1.60 ± 0.021	1.65 ± 0.022	1.64 ± 0.010	0.117	
	GM	1.64 ± 0.012	1.56 ± 0.051	1.67 ± 0.051	0.238	0.192
	*P* value	0.190	0.167	0.221		
CLA *cis*-9 *trans*-11	IS	0.90 ± 0.011	0.92 ± 0.011	0.90 ± 0.012	0.142	
	GM	0.93 ± 0.051	0.96 ± 0.032	0.94 ± 0.043	0.134	0.219
	*P* value	0.141	0.132	0.113		
CLA *trans*-10 *cis*-12	IS	0.90 ± 0.038	0.89 ± 0.005	0.91 ± 0.021	0.195	
	GM	0.82 ± 0.016	0.84 ± 0.006	0.85 ± 0.048	0.175	0.223
	*P* value	0.100	0.213	0.163		
C18:2n-6	IS	13.25 ± 0.120^a^	12.00 ± 0.110^b^	11.05 ± 0.761^c^	<.0001	
	GM	12.06 ± 0.140^a^	11.09 ± 0.120^b^	10.21 ± 0.111^c^	0.034	0.145
	*P* value	0.223	0.154	0.170		
C18:3n-3	IS	2.00 ± 0.050^a^	1.53 ± 0.030^b^	1.02 ± 0.010^c^	<.0001	
	GM	1.98 ± 0.020^a^	1.64 ± 0.010^b^	1.21 ± 0.031^c^	<.0001	0.134
	*P* value	0.210	0.130	0.230		
C20:4n-6	IS	5.40 ± 0.111^a^	4.47 ± 0.115^b^	4.38 ± 0.091^b^	<.0001	
	GM	5.19 ± 0.132^a^	4.60 ± 0.119^b^	4.00 ± 0.145^c^	<.0001	0.145
	*P* value	0.101	0.219	0.231		
C20:5n-3	IS	1.11 ± 0.010^a^	1.02 ± 0.020^ab^	0.78 ± 0.031^b^	<.0001	0.250
	GM	1.40 ± 0.021^a^	1.37 ± 0.030^a^	1.02 ± 0.012^b^	0.024	
	*P* value	0.142	0.133	0.212		
C22:5n-3	IS	1.20 ± 0.041^a^	1.00 ± 0.010^b^	0.90 ± 0.020^c^	0.041	
	GM	1.31 ± 0.022^a^	1.03 ± 0.020^b^	1.00 ± 0.031^c^	0.023	0.461
	*P* value	0.072	0.221	0.090		
C22:6n-3	IS	0.90 ± 0.010^a^	0.80 ± 0.022^b^	0.75 ± 0.005^b^	<.0001	
	GM	0.91 ± 0.021^a^	0.83 ± 0.021^b^	0.72 ± 0.008^c^	0.004	0.220
	*P* value	0.221	0.316	0.145		
Intramuscular fat (mg/100 g)	IS	3030.23 ± 20.231^y^	3100.15 ± 30.001^y^	3050.00 ± 20.331^y^	0.110	
	GM	2880.90 ± 10.122^x^	2840.69 ± 20.450^x^	2890.21 ± 15.211^x^	0.108	0.211
	*P* value	0.042	0.040	0.041		
∑SFA	IS	42.72 ± 0.451^c^	45.81 ± 0.141^b^	47.74 ± 0.332^a^	<.0001	
	GM	43.41 ± 0.230^c^	46.27 ± 0.220^b^	48.35 ± 0.331^a^	0.001	0.112
	*P* value	0.126	0.327	0.114		
∑MUFA	IS	31.43 ± 0.110	31.85 ± 0.420	31.98 ± 0.610	0.212	
	GM	32.00 ± 0.191	31.37 ± 0.291	32.14 ± 0.171	0.450	0.341
	*P* value	0.116	0.221	0.145		
∑PUFA	IS	25.66 ± 0.547^a^	22.73 ± 0.219^b^	20.69 ± 0.425^c^	0.001	
	GM	24.60 ± 0.226^a^	22.36 ± 0.137^b^	19.95 ± 0.289^c^	0.012	0.217
	*P* value	0.112	0.651	0.231		
∑n-3	IS	5.21 ± 0.021^a^	4.35 ± 0.017^b^	3.45 ± 0.011^c^	0.004	
	GM	5.60 ± 0.050^a^	4.87 ± 0.018^b^	3.95 ± 0.010^c^	0.001	0.431
	*P* value	0.230	0.112	0.349		
∑n-6	IS	18.65 ± 0.292^a^	16.57 ± 0.440^b^	15.43 ± 0.115^c^	0.001	
	GM	17.25 ± 0.451^a^	15.69 ± 0.225^b^	14.21 ± 0.389^c^	0.003	0.144
	*P* value	0.932	0.238	0.476		
n-6:n-3	IS	3.57 ± 0.011^cy^	3.80 ± 0.017^by^	4.47 ± 0.016^ay^	0.001	0.221
	GM	3.08 ± 0.022^cx^	3.22 ± 0.008^bx^	3.59 ± 0.025^ax^	0.001	
	*P* value	0.045	0.032	0.023		
UFA:SFA	IS	1.34 ± 0.017	1.19 ± 0.010	1.10 ± 0.007	0.779	0.100
	GM	1.33 ± 0.028	1.16 ± 0.020	1.08 ± 0.039	0.174	
	*P* value	0.212	0.661	0.132		
PUFA:SFA	IS	0.60 ± 0.016 ^a^	0.49 ± 0.010^b^	0.43 ± 0.020^c^	0.009	
	GM	0.56 ± 0.028 ^a^	0.47 ± 0.030 ^b^	0.41 ± 0.010^c^	0.032	0.109
	*P* value	0.142	0.182	0.112		

^a, b, c^ means having different superscript along the same row are significantly different (*P* < 0.05). ^x, y^ means having different superscript along the same column are significantly different (*P* < 0.05). ∑SFA = (C14:0 + C15:0 + C16:0 + C17:0 + C18:0), ∑MUFA = (C14:1 + C15:1 + C16:1+ C17:1 + C18:1+ C18:1 *trans*-11), ∑UFA = (∑MUFA + C18:1 *trans*-11+ CLA cis-9 *trans*-11+ CLA cis-12 *trans*-10 + ∑n-3 + ∑n-6), ∑PUFA = (CLA cis-9 *trans*-11+ CLA cis-12 *trans*-10 + ∑n-3 + ∑n-6), ∑n-3 = (C18:3n-3 + C20:5n-3 + C22:5n-3 + C22:6n-3), ∑n-6 = (C18:2n-6 + C20:4n-6) n-6:n-3 = (C18:2n-6 + C20:4n-6) ÷ (C18:3n-3 + C20:5n-3 + C22:5n-3 + C22:6n-3), UFA:SFA = (∑UFA)/∑SFA), PUFA:SFA = (∑PUFA/∑SFA)

The major fatty acids in the muscles were C18:1n-9, C18:0 and C16:0. Similar findings were observed in beef [68], chevon [69] and mutton [70]. Muscle type had no effect (*P* > 0.05) on the FA composition. However, GM had lower (*P* < 0.05) n6/n3 ratio than IS throughout storage. *Postmortem* chill storage had a significant (*P* < 0.05) impact on the concentration of some FA in both muscles. The concentration of all n-3 and n-6 FA, and total polyunsaturated fatty acids (PUFA) decreased (*P* < 0.05) while the concentration of individual saturated fatty acids (SFA) and total SFA increased (*P* < 0.05) as storage progressed. This observation concurs with that of Muíño et al. [70] who observed a decrease in the concentration of n-3 and n-6 PUFA in mutton aged for 12 d. Similarly, the concentration of n-3 and n-6 FA decreased during a 7 d chilled storage of mutton [71]. Except C16:1n-7 and C17:1 whose concentration increased (*P* < 0.05) over storage, most monounsaturated fatty acids (MUFA) were unaffected (*P* > 0.05) by *postmortem* chill storage. The instability of PUFA coincides with the increase in TBARS values and the reduction in the concentration of carotenoid and tocopherol as storage progressed. The reduction in the concentration of PUFA was responsible for the increment in the concentration of SFA. The concentration of CLA *cis*-9 *trans*-11 and CLA *trans*-10 *cis*-12 and total MUFA was stable (*P* > 0.05) throughout storage. This indicates that CLA and MUFA have less propensity to oxidize compared with PUFA. There was no significant interaction between muscle type and *postmortem* chill storage for IMF and fatty acid composition of goat meat.

## Conclusion

The results of this study evince that changes in meat quality during *postmortem* chill storage is inexorable. However, muscles differ in their response to *postmortem* chill storage. The IS muscle had higher redness and lower cooking loss on d 0 and 1, and lower shear force on d 0, 1 and 4 than GM. However, IS had greater drip loss, carbonyl content, MHC degradation and colour deterioration than GM on d 7. The greater SOD and CAT activities in IS suggests a feedback mechanism in response to the greater oxidative deterioration. The GM had lower colour deterioration and MHC degradation and thus suitable for chilled storage for longer periods. The MHC and colour of IS could be maintained for up to 4 days of *postmortem* chill storage. The tocopherol and carotenoids seemed to be the primary drivers for *postmortem* oxidative stability of goat meat during chill storage. Further study to examine the effect of *postmortem* chill storage for extended periods on the antioxidant status, degradation of myofibrillar proteins, physicochemical properties and microbiological quality of different muscles in goat is suggested.
